# Transition phase of nutrition—optimizing nutrient administration

**DOI:** 10.3389/fped.2025.1658550

**Published:** 2025-09-05

**Authors:** Pradeep Alur, Sumana Ramarao

**Affiliations:** ^1^ Department of Pediatrics, Penn State College of Medicine, Penn State Health, Hampden Medical Center, Enola, PA, United States; ^2^ Department of Pediatrics, University of Mississippi Medical Center, Jackson, MS, United States

**Keywords:** nutrition, transition phase, VLBW (very low birth weight), preterm infant, parenteral nutrition

## Abstract

This discussion explores the complex aspects of managing nutrition for preterm infants during the critical transition from exclusive parenteral nutrition (PN) to full enteral feeding (EN). The primary objectives of nutritional care in this very low birth weight infants (VLBW) population are to promote growth rates comparable to those *in utero* and enhance key neurodevelopmental milestones. The transition phase is characterized by the gradual increase of enteral feeds concurrently with the reduction and eventual cessation of parenteral nutrition. This period presents several key challenges in clinical practice, marked by notable variability: (A) determining the appropriate timing and criteria for initiating enteral feeds; (B) optimizing the rate at which enteral feed volumes are safely increased; (C) deciding the specific enteral volume threshold when to initiate human milk fortification to meet increasing caloric and protein demands; (D) establishing the optimal timing for discontinuing intravenous lipid emulsions (ILE); and (E) identifying the precise enteral volume threshold that dictates when to cease parenteral amino acid administration. To navigate these complexities and ensure seamless nutrient administration, practical recommendations for effective management are crucial. These include advocating for early fortification of human milk, judicious use of concentrated parenteral nutrition to provide adequate nutrients in lower fluid volumes, and strategically minimizing the overall duration of the transition phase. Implementing these evidence-informed steps aims to ensure smooth nutrition management, optimize nutrient delivery, and significantly reduce the pervasive risk of postnatal growth failure in extremely low birth weight infants.

## Introduction

Substantial improvements in the survival rates of preterm infants have highlighted the ongoing complexities of their nutritional management, which is significantly influenced by gestational age, postmenstrual age (PMA), and inherent physiological immaturity. Small preterm infants often face feeding intolerance due to gut immaturity, manifesting as gastric dysmotility and intestinal hypomotility, alongside other complicating conditions. This challenge can slow the progression toward complete enteral nutrition. Consequently, parenteral nutrition is crucial in bridging the nutritional gap while enteral feeds gradually increase. This combined approach ensures adequate nutrient delivery to meet the infant's essential needs, ultimately supporting growth and improving health outcomes (1). Key objectives are to achieve extrauterine growth rates comparable to those *in utero*, promote gut maturation, and optimize immediate and long-term health outcomes, including neurodevelopment (2–5). Simultaneously, careful nutritional modulation is required to mitigate the risks of excessive postnatal weight gain and potential long-term sequelae such as obesity, diabetes, and cardiovascular disorders (6, 7). This careful balance extends to macronutrient provision; for instance, studies suggest excessive protein administration early in life may be associated with adverse neurodevelopmental outcomes (8), underscoring the critical need to provide sufficient protein for growth while avoiding potential harm from excess**.**

For many preterm infants, particularly those born at less than 32 weeks' gestation, nutritional support typically commences with exclusive parenteral nutrition (PN). Initial management often targets a fluid intake of approximately 100 ml/kg/day, providing 46–56 kcal/kg/day. Caloric delivery via PN is subsequently advanced over the following days, with a goal of 110–130 kcal/kg/day, to adequately support extrauterine growth and meet the high metabolic demands of this population.

## Nutrition guidelines

Current nutritional guidelines offer specific recommendations for distinct feeding stages in preterm infants. For instance, the American Academy of Pediatrics (AAP) (Table 1) recommends a minimum parenteral nutrition (PN) intake of 90 kcal/kg/day for extremely low birth weight (ELBW) infants (9). Once full enteral nutrition (EN) is achieved, caloric targets typically increase to 110–130 kcal/kg/day, often corresponding to fluid volumes approaching 150 ml/kg/day.

**Table 1:** Recommendations for parenteral and enteral nutrient intake in VLBW infants.

Nutrition	AAP	ESPGHAN
Parenteral Fluids (ml/kg/day)	100–160	80–100
Enteral Fluids (ml/kg/day)	135–200	150–180
Parenteral Energy (kCal/kg/day)	90–115	45–120
Enteral Energy (kCal/kg/day)	110–130	115–140
Parenteral Protein (g/kg/day)	1.5–3.5	1.5–3.5
Enteral Protein (g/kg/day)	3.5–4.0	3.5–4.0
Parenteral Lipids (g/kg/day)	1–3.0	1–4.0
Enteral Lipids (g/kg/day)	4.55–8.1	4.8–8.1

Despite these established benchmarks for exclusive PN and full EN, specific, evidence-based guidelines for managing nutrition during the transition phase are notably lacking, especially for ELBW infants. This transition phase is generally defined as the period when exclusive PN is slowly reduced and eventually stopped as EN volumes gradually increase.

However, a lack of agreement continues regarding the specific enteral feeding volumes that define the initiation and cessation of this critical phase. While the ultimate goal is often to reach full enteral feeds (e.g., ∼150 ml/kg/day), the transition occurs within intermediate volumes. For instance, Wang et al., in a systematic review supporting this need for definition, proposed a quantitative operational definition, suggesting the transition phase could encompass the period when minimum enteral feeding volumes increase from 30 ml/kg/day up to approximately 120 ml/kg/day (10). Nevertheless, significant variation regarding this upper threshold remains evident in clinical practice and the literature. The phase of nutrition when PN is combined with advancing enteral nutrition from 30 ml/kg/day to 120 ml/kg/day is considered the transitional phase of nutrition.

## Practice challenges

Research on the transition from parenteral to enteral nutrition in preterm infants consistently underscores the need for well-defined, evidence-based nutrient targets during this critical period. Managing nutrition in these infants is complicated by considerable variability in clinical practices worldwide. Key areas where these practices often differ include:

1. *The timing and criteria for initiating enteral feeds*:

Significant variation exists in practices for initiating enteral feeds, including the timing and whether a trophic feeding phase precedes advancement. In many centers, starting enteral feedings very early, often within 12 h. of birth, is standard practice. Administering the mother's own milk in sequence, starting with the initially produced colostrum, offers significant benefits because colostrum is uniquely rich in valuable bioactive compounds (1). Early progressive feeding, started without a trophic phase, was investigated in a single-center randomized controlled trial of 60 extremely preterm infants (<1,000 g). The study found that this approach increased infants' time on full enteral feeds and decreased their need for total parenteral nutrition (PN) without raising the incidence of other health complications (11). However, if a center practices trophic feeding, this initial non-nutritive phase can prolong PN duration and delay the time to reach complete enteral nutrition.

2. *The rate at which enteral feed volumes are increased*:

Historically, it was believed that slower advancement of smaller enteral volumes could help protect against Necrotizing Enterocolitis (NEC), even though it delayed full feedings (12). However, current evidence does not support this cautious approach (13). A review of 10 randomized controlled trials (RCTs) involving 3,753 infants found no reduction in the risk of NEC or death when comparing slower feeding advancement rates of 15–20 ml/kg/day to faster rates of 30–40 ml/kg/day in preterm very low birth weight (VLBW) infants (13). Similarly, the SIFT RCT, which included 2,793 preterm infants, showed no significant difference in the incidence of late-onset sepsis, NEC, or survival without moderate-to-severe neurodevelopmental disabilities between advancement rates of 30 ml/kg/day and 18 ml/kg/day (14). If the rate of advancement of feeds is slow, it may prolong the PN phase and unfortified breast milk provision, thus compromising nutrient delivery.

3. *The specific enteral volume threshold determines when to start human milk fortification*:

It is clearly evident that human milk reduces NEC compared to preterm formulas (15). Hence, human milk is preferred as the primary source of enteral feeding in infants with very low birth weights. Since human milk alone does not meet the nutritional requirements of the growing preterm infant, it is recommended to fortify it to a 24-calorie/oz (16). However, the enteral volume at which such fortification occurs varies widely (17). Such variance can result in the suboptimal supply of nutrients. One systematic review compared early (enteral volume of 40 ml/kg/day or less) vs. late fortification (≥75 ml/kg/day) (18). No differences were found between groups for in-hospital growth, risk of NEC, feed intolerance, sepsis, or mortality. The Cochrane review also reached a similar conclusion (19). However, one study noted that cumulative protein intake was higher with early fortification (EF) (20). The cumulative protein intake (g/kg) in the first 4 weeks of life was higher in the EF group [98.6 [93.8, 104] vs. 89.6 [84.2, 96.4], *P* < 0.001]. Therefore, the timing of human milk fortification is essential to ensure optimal provision of nutrients. Moreover, the fortification, standard, or individualized fortification may also impact nutrient delivery (21). By enhancing protein intake and supporting better somatic and head growth, adjustable fortification represents a practical approach to optimizing the nutritional value of fortified human milk.

4. *The timing for discontinuing intravenous lipid emulsions (ILE)*:

The American Academy of Pediatrics' nutrition recommendation for preterm infants does not provide optimal timing for discontinuing parenteral lipids. The recommended enteral fat provision for ELBW is about 4.5–8 g/kg/day (22). It is suggested that at least 0.5 g/kg/day of lipids is needed to prevent essential fatty acid deficiency (1). Fortified human milk provides approximately 4.8 g/kg/day of fat at a volume of 100 ml/kg (23). Hence, reducing the intravenous lipids to 1 g/kg/day when the fortified human milk volume is at 80 ml/kg/day and discontinuing the intravenous lipids at 100 ml/kg/day of enteral volume of fortified human milk is safe and provides the recommended fat provision. This suggested approach also highlights the importance of earlier human milk fortification.

5. *The enteral volume threshold dictates when to cease parenteral amino acid administration:*

Studies suggest that the gut and liver consume almost 40 to 50% of the amino acids during the first pass (24, 25). Therefore, premature discontinuation of parenteral amino acids may lead to an inadequate supply of protein in the VLBW infant. It is suggested that parenteral nutrition be weaned when the enteral volume reaches 75 ml/kg/day (25). However, the recommendation is unclear about whether the fortification of human milk is initiated before weaning from parenteral nutrition. The current recommendation from AAP is to discontinue parenteral nutrition once the enteral volume reaches 100–120 ml/kg/day.

Recognizing these varying approaches emphasizes the challenges of optimizing care. However, a thorough examination of the rationale and implications for each specific variation in practice exceeds the scope of this article.

## Transition phase studies

As there are specific guidelines for the parenteral and enteral phases of nutrition in infants with very low birth weight (VLBW), the provision of nutrients may remain consistent during these phases (26, 27). However, the absence of such recommendations during the transition phase of nutrition results in an inconsistent and highly variable approach to nutrition by neonatal caregivers. Miller et al. noted that overall growth was adequate during the parenteral and enteral phases of nutrition; however, growth was compromised during the transitional phase (28). Growth velocity of <10 g/kg/day was considered poor growth. The incidence of poor growth during the parenteral, enteral, and transition phases of nutrition was 22.5%, 17.1%, and 46.1%, respectively. The odds of poor growth during the transition phase of nutrition were 5.4 (95% CI: 1.66–17.52). In this study, the authors identified that growth was compromised during the transitional phase primarily due to reduced protein intake as the parenteral nutrition was weaned and enteral nutrition was introduced. The study recommends maintaining protein intake at or above 3 g/kg/day and early human milk fortification with enteral feeds at 80 ml/kg/day.

Brennan et al., in their observational study, demonstrated macronutrient and energy deficits in the transition phase of nutrition in <34 weeks preterm infants (29). The study emphasizes the implementation of a nutrition phase (PN, transition phase, and EN) rather than a chronological age (postmenstrual age) approach to identify nutrient deficits at each phase, particularly during the transition phase, when enteral feeds reach 80 ml/kg/day and early fortification of human milk is initiated.

A subsequent prospective study by the same research group used a nutrient model to identify optimal amino acid provision during the parenteral nutritional transition for preterm infants (BW ≤1,500 g, GA <34 weeks) (30). The model yielded an optimal target of 3.5 g of amino acids per 100 ml. However, the interpretation requires caution, as clarification is needed on whether this represents a target concentration within fluids or how it translates to the standard daily intake goal (g/kg/day). The stated aim of this target was to support adequate growth and prevent nutrient deficits.

In this study (30), the researchers also proposed strategies to boost protein intake during the transition, such as customized PN formulations and appropriate human milk fortification. Their protocol included a specific fluid calculation method: enteral feeds below 40 ml/kg/day did not contribute to the fluid totals used for adjusting PN rates. This was intended to maintain consistent parenteral fluid and nutrient delivery, particularly during the initiation of minimal enteral feeds.

In a separate study, Miller et al. conducted a retrospective comparative analysis (31) that implemented a targeted nutrition protocol. They compared a study group receiving concentrated PN within a restricted total fluid volume of 100 ml/kg/day to a control group with a higher total fluid volume of 140 ml/kg/day. Both groups aimed for similar nutritional goals, targeting an energy intake of 100–120 kcal/kg/day and a protein intake greater than 3 g/kg/day, with intravenous lipids providing less than 50% of the calories derived from PN.

The weight-for-age z scores at birth, one week old, and during the transition phase were similar between groups. The findings revealed that the study group (restricted fluids with concentrated PN) achieved significantly higher weight-for-age z-scores by the end of the transition phase compared to the control group, with this advantage persisting until 35 weeks postmenstrual age. However, the authors noted that the actual delivery of protein and calories often fell short of targets when infants received total enteral feed volumes between approximately 100–130 ml/kg/day. This shortfall was potentially linked to the protocol's threshold for initiating human milk fortification, which was delayed until enteral volumes reached 100–120 ml/kg/day.

A similar correlation of the critical role of protein during the transition phase was highlighted by Liotto and the group in their NICU (32). The authors noted that very low birth weight infants with adequate growth velocity of at least 15 g/kg/day had higher enteral protein intake during the main parenteral period and higher parenteral protein and energy intakes during the main enteral nutritional intake period, suggesting a careful adjustment of protein during the transition phase.

To understand the potential risk of poor growth in the transition nutrition phase in very low birth weight infants, Immeli and group conducted a retrospective cohort study (33). The study highlights that the prolonged transition phase from parenteral to enteral nutrition correlates with lower cumulative intakes of energy, protein, fat, and carbohydrates at 28 days of age. The study specifically demonstrated that a prolonged transition phase of over 12 days in VLBW infants resulted in significantly lower weight and head circumference z-score changes at term-equivalent age compared to those with a shorter transition phase of 7 days. The study also reported more negative associations with the transition duration in boys than in girls. Interestingly, in another retrospective study by Alur et al., female ELBW infants experienced a significant decrease in weight percentiles during the transition phase compared to males with similar calorie and protein intake (34).

## Our suggested approach to the transition phase

We designed a new approach to nutrient provision for ELBW infants, carefully considering the challenges previously outlined (25–32). Building on this foundation and the studies discussed above, we suggest a practical strategy for the transition phase (TP), which includes concentrating PN, fortifying human milk earlier, and reducing the overall duration of the TP (35):

(A) Parenteral nutrition is initiated immediately at birth with 3.5 g/kg/day of amino acids in preterm infants with birth weights of 1,000–1,500 grams and 2.5–3 g/kg/day in preterm infants with BW of <1,000 grams (1), and 100 ml/kg/day of PN.
(B) Additional fluids, such as low-concentration dextrose-containing fluids, may be provided to meet the insensible losses.
(C) Lipids are started at 1 g/kg/day and are advanced if the triglyceride concentrations are less than 265 mg/dl (36). The lipid fluid volumes are in addition to the PN fluid.
(D) Dextrose concentrations are modified to keep the blood glucose concentrations below 180 mg/dl.
(E) Trophic enteral feeds are initiated as soon as possible if hemodynamically stable at 20 ml/kg/day. The feeds are advanced at 20 ml/kg/day daily if the infant is stable until a total enteral feed volume of 150–160 ml/kg/day is achieved. The duration of the trophic feeds is based on the infant's clinical stability.
(F) The feeds are either mother's milk or donor breast milk. The feeds are fortified to 24 kcal/oz once an enteral feed volume of 50 ml/kg/day is achieved. AAP recommends as much human milk, either donor or mother's own, as possible before 34 weeks postconceptional age to reduce the risk of necrotizing enterocolitis. However, donor milk may have lower protein, immunoglobulins, and electrolyte content, and up to 30% reduction in fat absorption may account for poor postnatal growth (9)
(G) The choice between fortifying human milk with preterm formula powder vs. a commercial human milk fortifier (HMF) is complex. Although some evidence indicates similar growth outcomes (non-inferiority) between these methods (37), commercial HMFs are specifically formulated to complement the nutrient profile of human milk.
(H) Among individualized fortification strategies, adjustable fortification using a BUN (blood urea nitrogen) level of 10 mg/dl as the cutoff may be more convenient compared to targeted fortification, which requires breast milk analyzers and is labor-intensive. However, neither approach is superior to the other (38). Decisions are therefore typically multifactorial, guided by institutional guidelines, specific nutrient targets, product availability, cost, potential tolerance issues, and the infant's clinical condition. The PN is weaned as enteral feeds are advanced to keep total PN and enteral fluids at 150 ml/kg/day.

Monitoring the growth of preterm infants during their hospital stay is essential for early detection of extrauterine growth restriction (EUGR). Failing to meet expected growth rates can result in adverse health outcomes, including impaired neurodevelopment and increased morbidity (39, 40).

The total calories provided at 100 ml/kg/day of PN [Dextrose10%w and 3.5 g/kg/day of amino acids (AA)] and 50 ml/kg/day of 24 kcal/oz mother's milk (BM-24) or donor breast milk will be 88 kcal/kg/day, and with lipids at 3 g/kg/day will provide 118 kcal/kg/day (Table 2).

**Table 2:** Transition phase nutrition approach.

PN-Ml/kg/day	D10 + 3.5%AA-Calories	BM-24 cal/oz- calories	Lipids-3 g/kg-Calories	Total calories-Kcal/kg/day	Protein-g/kg/day
100	48	50 ml/kg- 40 cal	30	118	4.9
50	24	100 ml/kg- 80 cal	0	104	4.55
30	14	120 ml/kg/day- 96 cal	0	110	4.4

When enteral feeds are advanced to 100 ml/kg/day and lipids are discontinued, the PN will be at 50 ml/kg/day to provide a total calories of 104 kcal/kg/day. When the PN is discontinued at 120 ml/kg/day of the enteral volume of BM-24, the total calories provided will be 110 kcal/kg/day as noted (refer to the table with nutritional characteristics). The total combined protein would be between 4.4 and 4.9 g/kg/day. The higher protein provided during the transition phase may help mitigate the first-pass effect (24, 25, 41), and the consensus suggests that increasing enteral intake by up to 50% will have a minimal impact on the systemic availability of amino acids (24). Thus, throughout the transition phase, adequate calories and protein are provided.

We also strongly recommend that a randomized controlled trial of the transition phase of nutrition may help clarify the optimal approach.

## Conclusions

In summary, extremely low birth weight infants, in particular, and those who experienced fetal growth restriction, are at risk for poor postnatal growth and require tailored nutritional interventions (38). Hence, neonatal caregivers should pay careful attention to the five challenges outlined: timing of initiating enteral feeds, rate of advancement of enteral feed volumes, enteral volumes at which human milk fortification is introduced, timing of discontinuation of ILE, and enteral volumes at which PN is discontinued for streamlining the nutrient delivery during TP.

Addressing these challenges with diligence is crucial for streamlining nutrient delivery. Our proposed TP phase policy offers a framework for developing robust TP nutrition guidelines.

Every neonatal unit caring for ELBW infants needs to create its specific nutritional guidelines for the transition phase. This ensures a seamless shift from parenteral to enteral nutrition and helps minimize postnatal growth failure. We strongly advocate for multicenter prospective trials to identify the most effective nutritional approaches during this critical period.
